# Emerging Human Infectious Diseases: Anthroponoses, Zoonoses, and Sapronoses

**DOI:** 10.3201/eid0903.020208

**Published:** 2003-03

**Authors:** Zdenek Hubálek

**Affiliations:** * Academy of Sciences, Brno, Czech Republic

**To the Editor**: The source of infection has always been regarded as an utmost factor in epidemiology. Human communicable diseases can be classified according to the source of infection as anthroponoses (when the source is an infectious human; interhuman transfer is typical), zoonoses (the source is an infectious animal; interhuman transfer is uncommon), and sapronoses (the source is an abiotic substrate, nonliving environment; interhuman transfer is exceptional). The source of infection is often the reservoir or, in ecologic terms, the habitat where the etiologic agent of the disease normally thrives, grows, and replicates. A characteristic feature of most zoonoses and sapronoses is that once transmitted to humans, the epidemic chain is usually aborted, but the clinical course might be sometimes quite severe, even fatal. An ecologic rule specifies that an obligatory parasite should not kill its host to benefit from the adapted long-term symbiosis, whereas an occasionally attacked alien host, such as a human, might be subjected to a severe disease or even killed rapidly by the parasite because no evolutionary adaptation to that host exists (*1*). In this letter, only microbial infections are discussed; metazoan invasion and infestations have been omitted.

Anthroponoses (Greek “anthrópos” = man, “nosos” = disease) are diseases transmissible from human to human. Examples include rubella, smallpox, diphtheria, gonorrhea, ringworm (*Trichophyton rubrum*), and trichomoniasis.

Zoonoses (Greek “zoon” = animal) are diseases transmissible from living animals to humans (*2*). These diseases were formerly called anthropozoonoses, and the diseases transmissible from humans to animals were called zooanthroponoses. Unfortunately, many scientists used these terms in the reverse sense or indiscriminately, and an expert committee decided to abandon these two terms and recommended “zoonoses” as “diseases and infections which are naturally transmitted between vertebrate animals and man” (*3*). A limited number of zoonotic agents can cause extensive outbreaks; many zoonoses, however, attract the public’s attention because of the high death rate associated with the infections. In addition, zoonoses are sometimes contagious for hospital personnel (e.g., hemorrhagic fevers). Zoonotic diseases can be classified according to the ecosystem in which they circulate. The classification is either synanthropic zoonoses, with an urban (domestic) cycle in which the source of infection are domestic and synanthropic animals (e.g., urban rabies, cat scratch disease, and zoonotic ringworm) or exoanthropic zoonoses, with a sylvatic (feral and wild) cycle in natural foci (*4*) outside human habitats (e.g., arboviroses, wildlife rabies, Lyme disease, and tularemia). However, some zoonoses can circulate in both urban and natural cycles (e.g., yellow fever and Chagas disease). A number of zoonotic agents are arthropod-borne (*5*); others are transmitted by direct contact, alimentary (foodborne and waterborne), or aerogenic (airborne) routes; and some are rodent-borne.

Sapronoses (Greek “sapros” = decaying; “sapron” means in ecology a decaying organic substrate) are human diseases transmissible from abiotic environment (soil, water, decaying plants, or animal corpses, excreta, and other substrata). The ability of the agent to grow saprophytically and replicate in these substrata (i.e., not only to survive or contaminate them secondarily) are the most important characteristics of a sapronotic microbe. Sapronotic agents thus carry on two diverse ways of life: saprophytic (in an abiotic substrate at ambient temperature) and parasitic (pathogenic, at the temperature of a homeotherm vertebrate host). Typical sapronoses are visceral mycoses caused by dimorphic fungi (e.g., coccidioidomycosis and histoplasmosis), “monomorphic” fungi (e.g., aspergillosis and cryptococcosis), certain superficial mycoses (*Microsporum gypseum*), some bacterial diseases (e.g., legionellosis), and protozoan (e.g., primary amebic meningoencephalitis). Intracellular parasites of animals (viruses, rickettsiae, and chlamydiae) cannot be sapronotic agents. The term “sapronosis” was introduced in epidemiology as a useful concept (*6*–*8*). For these diseases the expert committee applied the term “sapro-zoonoses,” defined as “having both a vertebrate host and a nonanimal developmental site or reservoir (organic matter, soil, and plants)” (*3*,*9*). However, the term sapronoses is more appropriate because animals are not the source of infection for humans. While anthroponoses and zoonoses are usually the domains for professional activities of human and veterinary microbiologists, respectively, sapronoses may be the domain for environmental microbiologists. The underdiagnosis rate for sapronoses is probably higher than that for anthroponoses and zoonoses, and an increase should be expected in both incidence and number of sapronoses. Legionellosis, Pontiac fever, nontuberculous mycobacterioses, and primary amebic meningoencephalitis are a few sapronoses that have emerged in the past decade. In addition, the number of opportunistic infections in immunosuppressed patients has grown markedly; many of these diseases and some nosocomial infections are, in fact, also sapronoses.

As with any classification, grouping human diseases in epidemiologic categories according to the source of infection has certain pitfalls. Some arthropod-borne diseases (urban yellow fever, dengue, epidemic typhus, tickborne relapsing fever, epidemic relapsing fever, and malaria) might be regarded as anthroponoses rather than zoonoses because the donor of the infectious blood for the vector is an infected human and not a vertebrate animal. However, the human infection is caused by an (invertebrate) animal in which the agent replicates, and the term zoonoses is preferred. HIV is of simian origin with a sylvatic cycling among wild primates and accidental infection of humans who hunted or ate them; the human disease (AIDS) might thus have been regarded as a zoonosis in the very first phase but later has spread in the human population as a typical anthroponosis and caused the present pandemic. Similarly, pandemic strains of influenza developed through an antigenic shift from avian influenza A viruses. For some etiologic agents or their genotypes, both animals and humans are concurrent reservoirs (hepatitis virus E, Norwalk-like calicivirus, enteropathogenic *Escherichia coli*, *Pneumocystis*, *Cryptosporidium, Giardia*, and *Cyclospora*); these diseases might conditionally be called anthropozoonoses. Other difficulties can occur with classifying diseases caused by sporulating bacteria (*Clostridium* and *Bacillus*): Their infective spores survive in the soil or in other substrata for very long periods, though they are usually produced after a vegetative growth in the abiotic environment, which can include animal carcasses. These diseases should therefore be called sapronoses. For some other etiologic agents, both animals and abiotic environment can be the reservoir (*Listeria*, *Erysipelothrix*, *Yersinia pseudotuberculosis, Burkholderia pseudomallei*, and *Rhodococcus equi*), and the diseases might be, in fact, called saprozoonosis (not sensu 9 ) in that their source can be either an animal or an abiotic substrate.

For a concise list of anthropo-, zoo-, and sapronoses, see the Appendix.

## Supplementary Material

Appendix Important Anthroponoses, Zoonoses, and Sapronoses
