# Supplementary material for: Emerging Human Infectious Diseases: Anthroponoses, Zoonoses, and Sapronoses
Source: Emerg Infect Dis. 2003 Mar;9(3):403–4. doi: 10.3201/eid0903.020208 (PMC2958532; doi:10.3201/eid0903.020208)
Supplement: Appendix — Important Anthroponoses, Zoonoses, and Sapronoses [file 02-0208_app-s1.pdf]

Appendix: **Important Anthroponoses, Zoonoses, and Sapronoses**[1]

Anthroponoses

Measles*; rubella; mumps; influenza; common cold; viral hepatitis; poliomyelitis; AIDS*; infectious mononucleosis; herpes simplex; smallpox; trachoma; chlamydial pneumonia and cardiovascular disease*; mycoplasmal infections*; typhoid fever; cholera; peptic ulcer disease*; pneumococcal pneumonia; invasive group A streptococcal infections; vancomycin-resistant enterococcal disease*; meningococcal disease*; whooping cough*; diphtheria*; *Haemophilus* infections* (including Brazilian purpuric fever*); syphilis; gonorrhea; tuberculosis* (multidrug-resistant strains); candidiasis*; ringworm (*Trichophyton rubrum*); *Pneumocystis* pneumonia* (human genotype); microsporidial infections*; cryptosporidiosis* (human genotype); giardiasis* (human genotype); amebiasis; and trichomoniasis.

Zoonoses Transmitted by Direct Contact, Alimentary (Foodborne and Waterborne), or Aerogenic (Airborne) Routes

Rabies; hemorrhagic fever with renal syndrome*; hantavirus pulmonary syndrome*; Venezuelan*; Brazilian*; Argentinian and Bolivian hemorrhagic fevers; Lassa; Marburg; and Ebola hemorrhagic fevers*; Hendra and Nipah hemorrhagic bronchopneumonia*; hepatitis E*; herpesvirus simiae B infection; human monkeypox*;Q fever; sennetsu fever; cat scratch disease; psittacosis; mammalian chlamydiosis*; leptospirosis; zoonotic streptococcosis; listeriosis; erysipeloid; campylobacterosis*; salmonellosis*; hemorrhagic colitis*; hemolytic uremic syndrome*; yersiniosis; pseudotuberculosis; sodoku; Haverhill fever; brucellosis*; tularemia*; glanders; bovine and avian tuberculosis*; zoonotic ringworm; toxoplasmosis; and cryptosporidiosis* (calf genotype 2).

Zoonoses Transmitted by Hematophagous Arthropods

Hard ticks (***Ixodidae***)

Russian spring-summer encephalitis; Central European encephalitis; louping ill; Kyasanur Forest disease; Powassan; Crimean-Congo hemorrhagic fever*; Colorado tick fever; Rocky Mountain spotted fever; boutonneuse fever; African tick typhus*; other rickettsial fevers*; human granulocytic ehrlichiosis*; Lyme disease*; tularemia; and babesiosis.

Soft ticks (***Argasidae***)

Tickborne relapsing fever

Mites (***Trombiculidae, Dermanyssidae***)

Scrub typhus; rickettsialpox

Lice (***Anoplura***)

Epidemic typhus; trench fever*; and epidemic relapsing fever

Triatomine Bugs (***Triatominae***)

Chagas disease

Sandflies (***Phlebotominae***)

Sandfly fever; vesicular stomatitis; Oroya fever; and leishmaniasis

Mosquitoes (***Culicidae***)

Eastern; Western; and Venezuelan equine encephalomyelitis; Sindbis fever; Chikungunya and O'nyong nyong fevers*; Ross River epidemic polyarthritis*; Japanese encephalitis*; West Nile fever*; St. Louis encephalitis; yellow fever; dengue/dengue hemorrhagic fever*; Murray Valley encephalitis; California encephalitis; Rift Valley fever*; and malaria*

Biting Midges (***Ceratopogonidae***)

Oropouche fever; vesicular stomatitis

Tsetse-flies (***Glossinidae***)

African trypanosomiasis

Fleas (***Siphonaptera***)

Murine typhus*; cat-scratch fever*; plague

Sapronoses

Chlamydia-like pneumonia* (amoebic endosymbionts *Parachlamydia acanthamoebae* and other *Parachlamydiaceae*); tetanus; gas gangrene (*Clostridium perfringens; C. septicum; C. novyi*); intestinal clostridiosis* (*C. difficile*; *C. perfringens*); botulism; food poisoning* (*Bacillus cereus*); anthrax; vibrio gastroenteritis* or dermatitis (*Vibrio parahaemolyticus; V. vulnificus*); nosocomial *Klebsiella pneumoniae* and *Pseudomonas aeruginosa* bacteremia* (including antibiotic-resistant strains); bacterial infections associated with cystic fibrosis* (*Burkholderia cepacia*; *Ralstonia* spp.); melioidosis* (*B. pseudomallei*); legionellosis* and Pontiac fever* (*Legionella pneumophila; L. micdadei*; and other spp.); atypical bacterial meningitis and sepsis* (*Chryseobacterium meningosepticum*); acinetobacter bacteremia* (*Acinetobacter calcoaceticus; A. baumannii; A. radioresistens*); corynebacterial endocarditis* (*Corynebacterium serosis; C. amycolatum* and other nondiphtheriae corynebacteria); rhodococcosis* (*Rhodococcus equi*); possibly leprosy (some strains of *Mycobacterium leprae* were detected as living saprophytically in wet moss habitats); Buruli ulcer disease* (*M. ulcerans*); mycobacterial diseases other than tuberculosis* (*M. kansasii; M. xenopi; M. marinum; M. haemophilum; M. fortuitum; M. scrofulaceum; M. abscessus;* and other spp.); nocardiosis (*Nocardia asteroides; N. brasiliensis*); actinomycetom (*Actinomadura madurae; A. pelletieri; Streptomyces somaliensis*); dermatophytosis (*Microsporum gypseum*); histoplasmosis*

(*Histoplasma capsulatum; H. duboisii*); blastomycosis (*Blastomyces dermatitidis*); emmonsiosis (*Emmonsia crescens; E. parva*); paracoccidioidomycosis (*Paracoccidioides brasiliensis*); coccidioidomycosis* (*Coccidioides immitis*); sporotrichosis (*Sporothrix schenckii*); cryptococcosis* (*Cryptococcus neoformans*); aspergillosis (*Aspergillus fumigatus*); mucormycosis (*Absidia corymbifera* and some other *Mucorales*); entomophthoromycosis (*Basidiobolus; Conidiobolus;* and *Entomophthora* spp.); maduromycetom (*Madurella mycetomatis; M. grisea; Pseudoallescheria boydii; Leptosphaeria senegalensis; Neotestudina rosatii*); chromoblastomycosis (*Phialophora verrucosa; Exophiala jeanselmei; Fonsecaea compacta; F. pedrosoi; Cladosporium carionii; Rhinocladiella aquaspersa*); phaeohyphomycosis (*Wangiella dermatitidis; Dactylaria gallopava; Exophiala spinifera*); fusariosis* (*Fusarium oxysporum; F. solani*); primary amebic meningoencephalitis* (*Naegleria fowleri*); and amoebic keratitis or chronic granulomatous amoebic meningoencephalitis* (*Acanthamoeba castellanii; A. polyphaga*).

[1] Emerging and reemerging diseases are marked with an asterisk.
